# Self-care and fatigue in individuals hospitalized with decompensated heart failure during the covid-19 pandemic: a cross-sectional study

**DOI:** 10.1590/1518-8345.7465.4617

**Published:** 2026-01-16

**Authors:** Laura da Silva Araujo, Kethlen Louise Palha Ferrari, Pedro Paulo Fernandes de Aguiar Tonetto, Daiane Vieira Medeiros Costa Zanetti, Carina Aparecida Marosti Dessotte, Rosana Aparecida Spadoti Dantas

**Affiliations:** 1 Universidade de São Paulo, Escola de Enfermagem de Ribeirão Preto, PAHO/WHO Collaborating Centre for Nursing Research Development, Ribeirão Preto, SP, Brazil. Universidade de São Paulo Escola de Enfermagem de Ribeirão Preto PAHO/WHO Collaborating Centre for Nursing Research Development SP Ribeirão Preto Brazil; 2 Scholarship holder at the Conselho Nacional de Desenvolvimento Científico e Tecnológico (CNPq), Brazil. Scholarship holder at the Conselho Nacional de Desenvolvimento Científico e Tecnológico Brazil; 3 Scholarship holder at the Coordenação de Aperfeiçoamento de Pessoal de Nível Superior (CAPES), Brazil. Scholarship holder at the Coordenação de Aperfeiçoamento de Pessoal de Nível Superior Brazil

**Keywords:** Self-Care, Fatigue, Heart Failure, COVID-19, Nursing, Cardiology

## Abstract

to determine the association between self-care and fatigue in patients hospitalized with decompensated heart failure during the COVID-19 pandemic.

observational, cross-sectional study involving 132 individuals hospitalized in a university hospital. Data were collected through individual interviews and medical record reviews. Self-care was evaluated using the Self-Care of Heart Failure Index, while fatigue was measured using the Fatigue Pictogram, both of which had been previously validated for use in Brazil. Scores of 70 or higher indicated adequate self-care in the domains of Management, Maintenance and Confidence.

most participants were men (n = 73; 55.3%), had a low educational level (n = 98; 74.2%), lived with a spouse and/or children (n = 77; 58.4%) and had a mean age of 62.3 years (standard deviation = 28.7). Inadequate self-care for heart failure was observed in the Maintenance (n = 109; 82.6%), Management (n = 81; 61.8%) and Confidence (n = 57; 48.3%) subscales. In the week before hospitalization, participants reported experiencing fatigue-related exhaustion and limitations. No statistically significant association was found between fatigue intensity or impact and the self-care subscales.

during the pandemic, although fatigue was prevalent among participants before hospitalization due to clinical decompensation of heart failure, it was not associated with self-care for the condition.

## Highlights

**(1)** Self-care for heart failure during was inadequate the pandemic. **(2)** Fatigue was a prevalent and debilitating symptom before hospitalization. **(3)** Nursing must prioritize self-care for heart failure.

## Introduction

Heart Failure (HF) is a chronic, multifactorial syndrome caused by structural or functional changes^(1)^ that compromise the heart’s ability to pump blood to meet tissue metabolic needs, resulting in reduced cardiac output^(2)^. The sudden or gradual onset of HF signs and symptoms, such as dyspnea, paroxysmal nocturnal dyspnea, orthopnea, arrhythmias, lower limb edema and fatigue, characterize the decompensation of the disease^(3)^. Self-care aimed at managing HF has proven essential in preventing disease decompensation, and is the focus of this study. We have adopted the definition of self-care proposed by Barbara Riegel and colleagues, namely the result of a decision-making process involving self-care maintenance, management and confidence behavior in people with HF^(4-5)^. Fatigue as a symptom has been associated with barriers to adherence to appropriate self-care behavior, as it causes physical and emotional difficulties, compromising an individual’s ability to care for themselves^(6-8)^.

Self-care is a key factor in leading a healthy lifestyle and managing signs and symptoms^(9)^. In the case of HF, it contributes to disease control, improves quality of life, reduces hospital admissions due to clinical decompensation and lowers healthcare costs^(10-13)^.

The relevance of investigating self-care is based on current review studies highlighting the need for interventions to encourage self-care among individuals with HF^(11,14)^. In clinical practice, nursing plays a crucial role in health education. The literature reports the use of different teaching strategies, the most common being personalized guidance based on inferior vena cava ultrasound, phone consultations, distribution of pamphlets and the formation of educational groups^(10,15)^. However, there is a need for greater investments to improve self-care behavior, reduce the number of unplanned hospital readmissions and maintain the quality of life of these individuals^(15-19)^. Patients’ knowledge about the disease contributes to understanding health guidelines and to their clinical monitoring at various levels of care^(19)^.

Throughout the course of HF, adjustments and adaptations in self-care planning may be necessary, for instance, in periods of disease exacerbation, development of new comorbidities or situations requiring more advanced therapies. During the COVID-19 pandemic, the health of individuals diagnosed with HF worsened, leading to greater exacerbation of the existing clinical condition, worse prognosis and higher mortality risk among those infected by SARS-CoV-2^(21-24)^. Self-care initiatives gained importance during the pandemic, aiming to prevent hospitalizations due to heart disease decompensation as well as exposure to the new coronavirus^(25)^.

In other countries, such as France^(26)^ and the United States of America^(27-29)^, studies have evaluated self-care in individuals with HF during the pandemic. In Brazil, only one study has been identified so far^(30)^. The authors investigated patients who had been hospitalized for clinical decompensation and concluded that the lack of monitoring by healthcare providers impacted self-care management in those patients^(30)^. This study aimed to evaluate the association between self-care and fatigue in patients hospitalized with HF decompensation during the COVID-19 pandemic.

## Method

### Study design

Observational cross-sectional study^(31)^.

The study was reported according to guidance provided in Strengthening the Reporting of Observational Studies in Epidemiology (STROBE), recommended by The Enhancing the Quality and Transparency of Health Research Network (EQUATOR).

### Study site

The study was conducted in the inpatient units of the cardiology division at the *Hospital das Clínicas da Faculdade de Medicina de Ribeirão Preto da Universidade de São Paulo*, located in the interior of the state of São Paulo, Brazil. This is a public, university-affiliated, tertiary-level hospital linked to the Ribeirão Preto Medical School, University of São Paulo. It serves the population of Ribeirão Preto and other cities in the state of São Paulo, as well as individuals from other Brazilian states, and is the largest public hospital in the region.

### Accessible population and eligibility criteria of the sample

The accessible population consisted of patients who were admitted to the aforementioned hospital during the pandemic with a diagnosis of clinical decompensation of heart failure. Notably, the requirement for the occurrence of the clinical event for eligibility purposes has been confirmed in observational^(12,32-33)^ and intervention^(34-35)^ studies in nursing research. Thus, the final sample size was based on the occurrence of the clinical event, i.e., the hospitalization of patients due to clinical decompensation between May 2021 and October 2022. Therefore, it is a subset of the target population that was defined geographically and temporally^(31)^.

We investigated a non-probabilistic consecutive sample consisting of individuals who met the eligibility criteria: being 18 years or older, hospitalized in the aforementioned hospital between May 2021 and October 2022, and clinically stable and without fatigue and/or dyspnea at the time of the interview^(12,32,34)^.

To assess allopsychic orientation, the participants were asked six questions regarding their name, current date, day of week, place, age and city of residence. Those who answered more than two questions incorrectly were excluded^(32,35-36)^.

### Data collection

To identify potential participants, active and regular searches were conducted in the cardiology wards throughout the study period. Regular search made it possible to identify all patients hospitalized during the study. Data were obtained through individual interviews and from the participants’ electronic medical records. These included sociodemographic information, namely: gender, age, family monthly income, marital status, city of residence, education level (years of formal schooling) and employment status; and clinical information, namely: medications used, comorbidities, HF etiology, self-reported functional class, adapted from the New York Heart Association (NYHA), and left ventricular ejection fraction (LVEF), derived from echocardiographic examination and subsequently categorized as preserved (> 50%), intermediate (40 - 49%) and reduced (≤ 40%)^(3)^.

Self-care in HF was assessed with the Self-Care of Heart Failure Index (SCHFI), as adapted for Brazilian Portuguese. The reference period for answering the instrument’s items was the previous month, which made it possible to assess the participants’ self-care in the period preceding their hospitalization.

The instrument consists of 22 questions divided into three subscales: Self-Care Maintenance (10 items), Self-Care Management (6 items) and Self-Care Confidence (6 items). In the Maintenance subscale, responses for each item range from never/rarely (1) to always/daily (4), except for item 8, where the scores are reversed. In the Management subscale, responses range from unlikely (1) to very likely (4). In the Confidence subscale, responses range from not confident (1) to extremely confident (4). The sums of the scores for each subscale are then transformed into a scale from zero to 100, with higher scores indicating better self-care in the respective subscale. The results can also be interpreted categorically: scores above 70 points indicate adequate self-care, while scores equal to or below 70 indicate inadequate self-care^(5)^.

The SCHFI was chosen based on the validity and reliability results verified in the original version^(5)^ and in the version adapted for Brazil^(37)^. The construct validity of the Brazilian version was demonstrated by the correlation test results between its self-care measures and those from equivalent questions in the Brazilian version of the European Heart Failure Self-care Behavior Scale (r = − 0.51; p < 0.001), and by the confirmation of the three subscales in the original version, according to confirmatory factor analysis. Adequate internal consistency of its items was verified by the Cronbach’s alpha values of 0.40, 0.82, and 0.93, obtained respectively for the Maintenance, Management and Confidence subscales^(37)^. The Brazilian version was used in other studies prior to the pandemic^(10,12)^.

To evaluate fatigue, we used the Fatigue Pictogram^(38)^ as validated for Brazil, which consists of two questions. The first is “How tired did you feel over the last week?”, with the following response options: not tired at all; a little tired; moderately tired; very tired; and extremely tired. The second question is “To what extent does feeling tired prevent you from doing what you want to do?”, with the possible responses being: I can do everything I usually do; I can do almost everything I usually do; I can do some of the things I usually do; I only do what I have to do; and I can do very little. The responses are accompanied by two sets of figures to help assess the intensity (question 1) and impact (question 2) of fatigue. The Brazilian version was considered a valid and reliable measure for cancer patients^(39)^. Later, a study conducted with heart failure patients found an association between the functional classes of NYHA and the scores of the instrument’s questions, demonstrating its validity for evaluating fatigue in this group as well^(32)^.

As the reference time for the responses to the items on the self-care and fatigue instruments are, respectively, the previous month and the previous week, we instructed participants to consider these periods prior to their current hospitalization.

### Analysis of results and statistics

The data were processed and analyzed descriptively using the Statistical Package for the Social Sciences, International Business Machines Corporation™ (IBM™ - SPSS) version 24.1 for Windows.

To test the association between self-care and fatigue, we used the Kruskal-Wallis test. The significance level adopted was 0.05.

### Ethical aspects

The study was conducted in accordance with national and international ethical guidelines and approved by the Research Ethics Committee of the University of São Paulo at Ribeirão Preto College of Nursing (CAAE No. 25497912.2.0000.5393). Informed consent was obtained from all individuals involved in the study through a written document.

## Results

During the study period, we recruited 137 potential participants. Of these, five were excluded due to lack of allopsychic orientation. The 132 participants had an average age of 62.3 years with a standard deviation (SD) of 28.7 years. The majority were men (55.3%), married or have a partner (58.3%), with up to nine years of schooling (74.2%), from other cities in the state of São Paulo (65.9%) and living with a spouse and/or children (58.4%). Regarding clinical characteristics, 59.1% had reduced LVEF and were in functional class IV, according to the NYHA (56.8%) (Table 1).

In the heart failure self-care measure, we found that the average scores were below the expected for adequate self-care (above 70) in the Maintenance and Management subscales. In the Maintenance subscale, the average score was 54.1, while in the Management subscale it was 60.6. In turn, the Confidence subscale reached the threshold score of 70. When we analyzed the breakdown of participants by adequate self-care, we found the following frequencies: 17.4% of participants in the Maintenance subscale, 38.2% in the Management subscale and 51.7% in the Confidence subscale (Table 2).

**Table 1-:** Sociodemographic and clinical characterization of the 132 participants. Ribeirão Preto, SP, Brazil, 2021-2022

**Sociodemographic and clinical characteristics**	n (%)*	**Mean (SD) ^†^**
**Age (years)**		62.3 (28.7)
**Sex**		
Male	73 (55.3)	
Female	59 (44.7)	
**Marital status**		
Married/Have a partner	77 (58.3)	
Single	24 (18.2)	
Widow(er)	18 (13.6)	
Separated/divorced	13 (9.8)	
**Educational level (years of schooling)**		5.9 (4.2)
Up to 9	98 (74.2)	
10-12	25 (18.9)	
13 or more	9 (6.8)	
**Monthly family income (in reais)**		2,397.88 (1,285.5)
Place of residence		
Other cities in the state of São Paulo	87 (65.9)	
Ribeirão Preto	41 (31.1)	
NYHA functional class ^‡^ (self-reported)		
Class 1	9 (6.8)	
Class 2	16 (12.1)	
Class 3	23 (17.4)	
Class 4	75 (56.8)	
**FEVE** ^§^		
Preserved	28 (21.2)	
Intermediate	12 (9.1)	
Reduced	78 (59.1)	
No information	14 (10.6)	

*n (%) = Number (percentage); ^†^SD = Standard deviation; ^‡^NYHA = New York Heart Association; ^§^LVEF = Left Ventricular Ejection Fraction

**Table 2-:** Self-care evaluation of the 132 participants according to the Self-Care Heart Failure Index. Ribeirão Preto, SP, Brazil, 2021-2022

SCHFI subscales*	**n (%) ^†^**	**Mean (SD) ^‡^**	**Median (minimum-maximum)**
**Maintenance (n=132)**		54.1 (18.0)	53.3 (3.3 – 96.6)
Adequate	23 (17.4)		
Inadequate	109 (82.6)		
**Management (n=131)**		60.6 (20.0)	65 (0 – 100)
Adequate	50 (38.2)		
Inadequate	81 (61.8)		
**Confidence (n=118)**		70 (19.1)	72.3 (22.2 – 100)
Adequate	61 (51.7)		
Inadequate	57 (48.3)		

*SCHFI = Self-Care of Heart Failure Index; ^†^n (%) = Number (percentage); ^‡^SD = Standard deviation

In the Maintenance subscale, the most frequently performed self-care items were: “attending doctor or nurse appointments regularly” (84.8%), “forgetting or missing to take medication” (reverse scores) (68.8%), “trying to avoid getting sick (e.g., getting a flu shot, avoiding contact with sick people)” (74.1%), “following a low-salt diet” (56.3%) and “checking if ankles are swollen” (48.2%). In turn, the items “exercising for 30 minutes” (75.9%), “engaging in physical activity” (66.1%) and “requesting low-salt foods when eating out or visiting someone” (61.6%) were answered with “never” or “rarely” by the participants.

In the Management subscale, the items “contacting doctor or nurse for guidance” (47.7%) and “reducing salt intake” (46.2%) were considered very likely to be done if participants experienced difficulty breathing or swollen ankles. The majority (90.2%) experienced breathing problems or swollen ankles in the past month, with 18.8% not recognizing these symptoms as related to heart failure; 29.9% recognizing them quickly; and 26.8% recognizing them immediately. Based on the resources provided in items two, three, four and five of the Management subscale, 28.6% of participants reported that they did not attempt any of the suggested resources the last time they had difficulty breathing or swollen ankles. Among those who tried any of the resources, 27.7% were totally sure that it helped, 23.2% were sure, 8.9% were not too sure and 9.8% were unsure.

For the Confidence subscale, the items with the highest confidence were: “following the recommended treatment” (45.5%), “assessing the importance of their symptoms” (42.0%) and “evaluating whether a medication works” (37.5%). The item with the lowest confidence was “being free from heart failure symptoms” (23.2%).

The Fatigue Pictogram showed that the majority of participants felt very tired (39.4%) or extremely tired (29.5%). The feeling of fatigue interfered with their daily activities and, consequently, with their quality of life, as 49.0% stated they could do very little or only what they had to do (20.5%) due to feeling debilitated (Table 3).

**Table 3-:** Fatigue evaluation of the 132 participants according to the Fatigue Pictogram. Ribeirão Preto, SP, Brazil, 2021-2022

**Fatigue Pictogram**	n (%)*
**Item 1 – How tired did you feel over the last week?**	
Not tired at all	9 (6.8)
A little tired	12 (9.1)
Moderately tired	20 (15.2)
Very tired	52 (39.4)
Extremely tired	39 (29.5)
**Item 2 – To what extent does feeling tired prevent you from doing what you want to do?**	
I can do everything I usually do	7 (5.3)
I can do almost everything I usually do	9 (6.8)
I can do some things I usually do	24 (18.2)
I only do what I have to do	27 (20.5)
I can do very little	65 (49.2)

*n (%) = Number (percentage)

The possible association between self-care measures and fatigue was evaluated, and no statistically significant results were found between intensity of fatigue and the Maintenance (p = 0.266), Management (p = 0.074) and Confidence (p = 0.059) subscales for self-care. Similar results were observed between impact of fatigue and the Maintenance (p = 0.834), Management (p = 0.609) and Confidence (p = 0.139) subscales for the participants’ self-care.

**Figure 1-:**
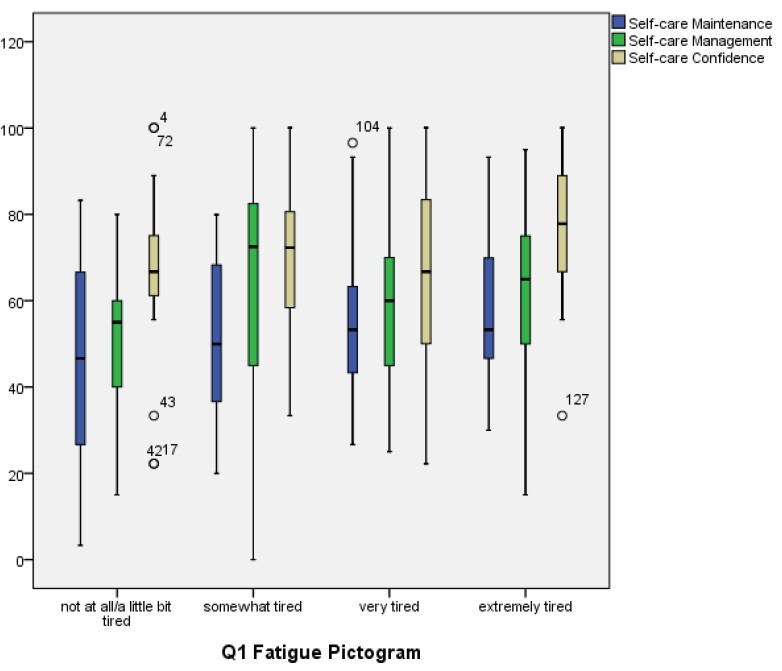
Boxplots with the results of the Maintenance, Management and Confidence self-care measures for heart failure according to intensity of fatigue

**Figure 2-:**
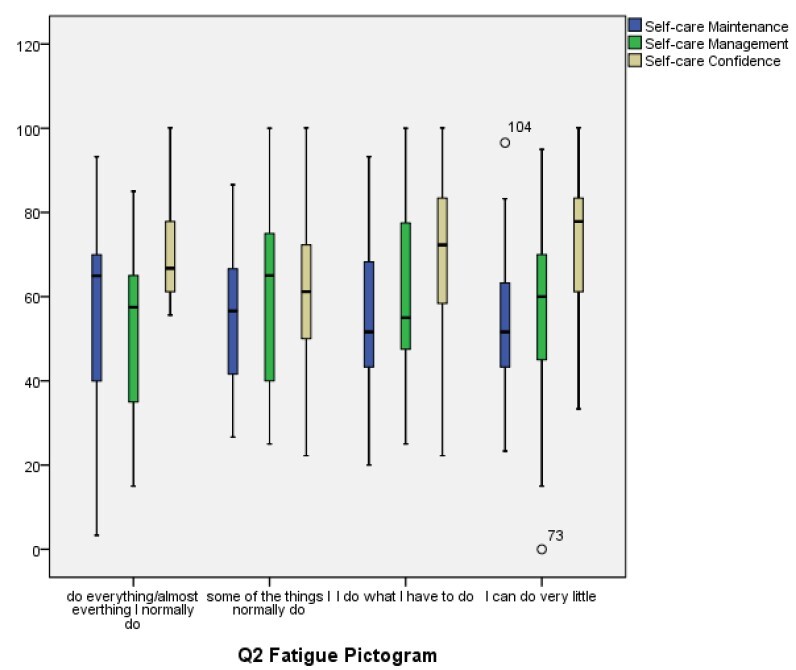
Boxplots with the results of the Maintenance, Management and Confidence self-care measures for heart failure according to impact of fatigue

The breakdown of the responses obtained in the Self-Care Maintenance, Self-Care Management and Self-Care Confidence subscales of the participants, according to intensity (question 1) and impact (question 2) of fatigue, are presented, respectively, in Figures 1 and 2.

## Discussion

The study aimed to examine the association between self-care and fatigue in patients hospitalized for heart failure decompensation during the COVID-19 pandemic. We found inadequate self-care maintenance and management in the month preceding hospitalization for clinical decompensation of heart failure. In the week leading up to hospitalization, most participants reported severe fatigue and compromised daily activities. However, no statistically significant associations were found between the self-care subscales (Maintenance, Management and Confidence) for heart failure and intensity of fatigue and its impact on daily activities.

Since this was an observational, cross-sectional study investigating a non-probabilistic sample of 132 patients, we consider the results to be exploratory in nature. The findings cannot be generalized to all patients with similar clinical characteristics who were hospitalized in Brazil during the COVID-19 pandemic. This sample is representative of the target population for the investigation, and although the participants were not randomly selected, they represent the population being studied. The results obtained, as well as those from other studies that used samples from clinical populations^(12,32-33)^, provide important contributions to the planning of intervention studies, as they will aid in calculating and determining sample size for these studies.

The interpretation of the results should be considered based on the epidemiological context in which the study was conducted, including the fact that the participants were recruited from a single healthcare facility (a public and teaching hospital in the interior of the state of São Paulo), the demographic characteristics of patients served by the Unified Health System, and, especially, the use of selected measurement instruments among a range of available options. The cross-sectional design of the study does not make it possible to infer cause-and-effect associations between fatigue and self-care in the studied sample. The World Health Organization’s declaration of the end of the global health emergency status for the COVID-19 pandemic will prevent the follow-up of the population of interest, for which a prospective study design could investigate the relationship between the two variables and clinical decompensation of heart failure.

During the COVID-19 pandemic, self-care behavior by people with HF decreased^(27-30)^, with difficulties in accessing healthcare services interfering with in-person monitoring of patients. In Brazil, poor adherence to pharmacological and dietary treatment were the main triggers of decompensated heart failure during the COVID-19 pandemic between March and October 2020^(40)^. With the limitation of appointments and fear of contagion^(20,25)^, patients with HF reduced their visits to healthcare services in Brazil^(41)^ and other countries^(20,23,25,42–44)^, which contributed to increased mortality^(44)^.

The sociodemographic profile of the participants, with most being men, older adults, married or in a domestic partnership, not employed in paid work, and with low income and low education, was also observed in other national and international studies^(30,32,41,45)^.

The results obtained should also be interpreted considering the selected measurement instruments for evaluating self-care in HF patients^(5,37)^ and the fatigue of individuals with this diagnosis^(38-39)^. Both scales enabled the comparison of results obtained during the pandemic with other investigations conducted before and during the COVID-19 pandemic.

When comparing the results obtained in the subscales of the SCHFI with those found by Brazilian researchers in studies conducted before the pandemic, we observed similar figures among patients seen in the outpatient clinic of the hospital where our study was conducted. These patients were followed-up during the first three months after discharge from hospitalization due to HF decompensation^(12)^. In another study, also carried out in a teaching hospital in São Paulo, similar results were reported by the researchers^(10)^. Our results were similar to those observed by researchers who used the same tool to assess self-care in patients in the United States of America^(27,29)^, Switzerland^(18)^, and China^(45)^.

Regarding fatigue symptoms, in the studied group, intensity of fatigue (very or extremely tired) and its impact on daily activities were reported by the participants. The presence of fatigue in people with HF is well documented^(32,46)^, affecting physical activities^(6)^, self-care^(6-8,47)^ and the quality of life of patients^(5,8)^. During the pandemic, physical activity decreased and symptoms of HF decompensation, such as fatigue, increased^(26)^. Individuals who had better symptom control before the pandemic maintained better self-care during the pandemic compared to those with poor self-care^(28)^. Although we found few studies that assessed fatigue using the same instrument^(6,32)^, our results corroborate those found in HF patients before the pandemic.

The results obtained corroborate those reported by other researchers and reinforce the need for nursing interventions focused on health literacy for the self-care of these individuals. Studies indicate that telemedicine played an important role during the pandemic, enabling monitoring, identification of priority patients and the provision of health guidance^(41,48-51)^. This kind of healthcare service had already been implemented before the pandemic; however, there was no consensus in studies regarding its effectiveness in patients with heart failure HF^(52)^. The pandemic was a factor that boosted its adoption, proving to be an efficient alternative^(26,28,52)^.

Nursing should consider self-care in HF as an active process to be planned together with patients and their family members, ranging from recognizing clinical decompensation symptoms to making the decision to seek healthcare. Thus, health education aims to achieve positive outcomes in adherence to both pharmacological and non-pharmacological treatments, supporting self-care management, maintenance and confidence for individuals with HF.

Regarding the contribution of this study to the advancement of scientific knowledge on the topic, we emphasize the existence of unsatisfactory self-care in HF in the four weeks preceding the need for unplanned hospitalizations due to clinical decompensation of the disease. When we compare the results of this study with those from previous ones, we observe that regardless of the COVID-19 pandemic, people with HF still need education focused on self-care for their health condition, with nurses being one of the most qualified healthcare providers to plan this complex intervention, not only for patients but also for their family members/caregivers.

The main limitations of the study were: the inability to calculate the sample size to determine the necessary number of participants to ensure the confirmatory nature of the results obtained; the cross-sectional design, which does not allow the researcher to infer causality between the variables, in this case, self-care and fatigue; and the recruitment of participants in a single hospital.

## Conclusion

During the COVID-19 pandemic, individuals hospitalized for clinical decompensation of heart failure assessed their self-care in the four weeks leading up to hospitalization as inadequate in terms of Management, Maintenance and Knowledge of heart failure. Fatigue was considered intense by most participants in the week prior to hospitalization, compromising their capacity to carry out daily activities. When examining the potential association between self-care and fatigue, we found no statistically significant results in the investigated group.

## Data Availability

Datasets related to this article will be available upon request to the corresponding author.
