# Supplementary material for: Self-care and fatigue in individuals hospitalized with decompensated heart failure during the covid-19 pandemic: a cross-sectional study
Source: Rev Lat Am Enfermagem. 2026 Jan 16;34:e4617. doi: 10.1590/1518-8345.7465.4617 (PMC12814791; doi:10.1590/1518-8345.7465.4617)
Supplement: Translation

# Translation: Autocuidado e fadiga em indivíduos internados com insuficiência cardíaca descompensada durante a pandemia da COVID-19: estudo transversal

**DOI:** 10.1590/1518-8345.7465.4618

**Authors:** Laura da Silva Araujo, Kethlen Louise Palha Ferrari, Pedro Paulo Fernandes de Aguiar Tonetto, Daiane Vieira Medeiros Costa Zanetti, Carina Aparecida Marosti Dessotte, Rosana Aparecida Spadoti Dantas

**Affiliations:** 1 Universidade de São Paulo, Escola de Enfermagem de Ribeirão Preto, Centro Colaborador da OPAS/OMS para o Desenvolvimento da Pesquisa em Enfermagem, Ribeirão Preto, SP, Brasil. Universidade de São Paulo Escola de Enfermagem de Ribeirão Preto Centro Colaborador da OPAS/OMS para o Desenvolvimento da Pesquisa em Enfermagem SP Ribeirão Preto Brasil; 2 Bolsista do Conselho Nacional de Desenvolvimento Científico e Tecnológico (CNPq), Brasil. Bolsista do Conselho Nacional de Desenvolvimento Científico e Tecnológico Brasil; 3 Bolsista da Coordenação de Aperfeiçoamento de Pessoal de Nível Superior (CAPES), Brasil. Bolsista da Coordenação de Aperfeiçoamento de Pessoal de Nível Superior Brasil

**Keywords:** Autocuidado, Fadiga, Insuficiência Cardíaca, COVID-19, Enfermagem, Cardiologia

## Abstract

verificar a associação entre autocuidado e fadiga de pacientes internados por descompensação da insuficiência cardíaca durante a pandemia da COVID-19.

estudo observacional, de corte transversal, com 132 indivíduos internados em um hospital universitário. Os dados foram coletados por entrevista individual e consulta aos prontuários. O autocuidado foi avaliado pelo *Self-Care of Heart Failure Index* e a fadiga pelo Pictograma de Fadiga, ambas versões validadas previamente para uso no Brasil. Valores iguais ou superiores a 70 indicam autocuidado adequado para as subescalas Manejo, Manutenção e Confiança.

a maioria dos participantes era do sexo masculino (n = 73; 55,3%), com baixa escolaridade (n = 98; 74,2%), morava com cônjuge e/ou filhos (n = 77; 58,4%) e a média de idade foi 62,3 anos (desvio-padrão = 28,7). Constatamos autocuidado inadequado para a insuficiência cardíaca nas subescalas Manutenção (n = 109; 82,6%), Manejo (n = 81; 61,8%) e Confiança (n = 57; 48,3%). Considerando a última semana antes da hospitalização, os participantes referiram cansaço e limitações decorrentes da fadiga. Não constatamos associação estatisticamente significante entre a intensidade e o impacto da fadiga e as subescalas do autocuidado.

no período pandêmico, a fadiga percebida pelos participantes antes da internação pela descompensação clínica da insuficiência cardíaca, embora prevalente, não teve associação com o autocuidado da doença.

## Destaques

**(1)** O autocuidado para insuficiência cardíaca foi inadequado durante a pandemia. **(2)** A fadiga foi um sintoma prevalente e incapacitante antes da internação. **(3)** O autocuidado para insuficiência cardíaca deve ser priorizado pela enfermagem

## Introdução

A insuficiência cardíaca (IC) é uma síndrome crônica multifatorial causada por alterações estruturais ou funcionais^(1)^ que comprometem a capacidade do coração em bombear sangue para atender às necessidades metabólicas tissulares, resultando na redução do débito cardíaco^(2)^. A manifestação súbita ou gradual dos sinais e sintomas da IC, tais como a dispneia, dispneia paroxística noturna, ortopneia, arritmias, edema de membros inferiores e fadiga, caracteriza a descompensação da doença^(3)^. O autocuidado voltado para o manejo da IC tem se mostrado fundamental para evitar a descompensação da doença, sendo o foco da investigação do estudo. Adotamos definição proposta por Bárbara Riegel e colaboradores, no sentido de que autocuidado se trata do resultado de um processo de tomada de decisão que envolve ações de manutenção, manejo e confiança para o autocuidado em pessoas com IC^(4-5)^. O sintoma fadiga tem sido associado às barreiras para adesão aos comportamentos adequados de autocuidado por promover dificuldade física e emocional, comprometendo a capacidade do indivíduo de se cuidar^(6-8)^.

O autocuidado é um fator fundamental para a adesão a um estilo de vida saudável e para o manejo dos sinais e sintomas^(9)^. No caso da IC, contribui para o controle da doença, melhora a qualidade de vida, reduz o número de internações devido à descompensação clínica e os custos dos serviços de saúde^(10-13)^.

A relevância da investigação do autocuidado baseia-se em estudos atuais de revisão sobre a necessidade de intervenções voltadas para o favorecimento do autocuidado de pessoas com IC^(11,14)^. No âmbito da prática clínica, a enfermagem apresenta papel fundamental na educação em saúde. Na literatura consta a utilização de diferentes estratégias de ensino, usadas de forma isolada ou conjunta, sendo as mais evidentes: orientação personalizada a partir do exame de ultrassonografia de veia cava inferior, consultas telefônicas, aplicação de folheto e formação de grupo educativo^(10,15)^. No entanto, há necessidade de maiores investimentos para melhorar o comportamento de autocuidado, diminuir o número de readmissões hospitalares não planejadas e manter a qualidade de vida desses indivíduos^(15-19)^. O conhecimento dos pacientes acerca da doença contribui para a compreensão das orientações em saúde e para seu acompanhamento clínico nos diversos níveis de cuidado^(19)^.

No decorrer da trajetória da IC, podem ser necessárias regulações e adaptações no planejamento do autocuidado, como, por exemplo, quando há período de agravamento da doença, desenvolvimento de novas comorbidades ou situações que necessitem de terapias mais avançadas. Durante a pandemia de coronavírus (COVID-19), constatou-se a piora das condições de saúde das pessoas com diagnóstico de IC, com maior agravamento do quadro clínico já existente^(20)^, piora do prognóstico e maior risco de mortalidade entre aqueles infectados pelo SARS-CoV-2^(21-24)^. Ações de autocuidado ganharam maior importância no período pandêmico, com vistas a evitar internações por descompensação da doença cardíaca, bem como pelo contágio com o novo coronavírus^(25)^.

Em outros países, como França^(26)^ e Estados Unidos da América^(27-29)^, foram realizados estudos que avaliaram o autocuidado de pessoas com IC durante a pandemia. No Brasil, apenas um estudo foi identificado até o momento^(30)^. Os autores investigaram pacientes que haviam sido hospitalizados por descompensação clínica e concluíram que a falta de acompanhamento por profissionais da saúde impactou na gestão do autocuidado desses pacientes^(30)^. O presente estudo teve o objetivo de verificar a associação entre autocuidado e fadiga de pacientes internados por descompensação da IC durante a pandemia da COVID-19.

## Método

### Delineamento do estudo

Estudo observacional, de corte transversal^(31)^.

O artigo foi reportado segundo os *Guidelines Strengthening the Reporting of Observational Studies in Epidemiology* (STROBE), recomendados pela *The Enhancing the Quality and Transparency of Health Research Network* (EQUATOR).

### Local do estudo

O estudo foi realizado nas unidades de internação da divisão de cardiologia do Hospital das Clínicas da Faculdade de Medicina de Ribeirão Preto da Universidade de São Paulo, localizado no interior do estado de São Paulo, Brasil. Trata-se de um hospital público e universitário, de nível terciário, vinculado à Faculdade de Medicina de Ribeirão Preto da Universidade de São Paulo. Atende à população de Ribeirão Preto e de outras cidades do estado de São Paulo, bem como de outros estados brasileiros, por ser o maior hospital público da região.

### População acessível e critérios de elegibilidade da amostra

A população acessível era composta por indivíduos internados no referido hospital durante a pandemia de COVID-19, com diagnóstico de descompensação clínica da IC. Cabe ressaltar que as pesquisas de enfermagem têm constatado a necessidade da ocorrência do evento clínico para a elegibilidade dos participantes, como se verifica tanto em estudos observacionais^(12,32-33)^ quanto de intervenção^(34-35)^. Assim, o tamanho final da amostra foi baseado na ocorrência do evento clínico, ou seja, a hospitalização de pacientes por descompensação clínica durante os meses de maio de 2021 a outubro de 2022. Trata-se, portanto, de um subconjunto da população-alvo que foi definido geograficamente e temporariamente^(31)^.

Investigamos uma amostra não probabilística, do tipo consecutiva, composta por aqueles indivíduos que atenderam aos critérios de elegibilidade: ter 18 anos ou mais; estar internado na instituição hospitalar supracitada durante o período de maio de 2021 a outubro de 2022; estar clinicamente estável, sem presença de fadiga e/ou dispneia no momento da entrevista^(12,32,34)^.

Para avaliar a orientação alopsíquica, foram feitas seis perguntas aos participantes sobre seu nome, data atual, dia da semana, localidade, idade e cidade de residência. Foram excluídos aqueles que responderam incorretamente mais de duas questões^(32,35-36)^.

### Coleta de dados

Para identificar os potenciais participantes, realizou-se uma busca ativa e regular nas enfermarias de cardiologia ao longo do período do estudo. A regularidade da busca permitiu identificar todos os pacientes internados durante a pesquisa. Os dados foram obtidos por entrevistas individuais e consultas aos prontuários eletrônicos dos participantes. Foram coletadas informações sociodemográficas, a saber: sexo, idade, renda mensal familiar, situação conjugal, cidade onde residia, escolaridade (anos que frequentou ensino formal) e situação profissional e clínica, a saber: medicamentos usados, comorbidades, etiologia da IC, classe funcional autorreferida, adaptada da *New York Heart Association* (NYHA), e fração de ejeção do ventrículo esquerdo (FEVE), proveniente do exame ecocardiográfico e, posteriormente, categorizada em preservada (> 50%), intermediária (40-49%) e reduzida (≤ 40%)^(3)^.

O autocuidado na IC foi avaliado pelo *Self-care of Heart Failure Index* (SCHFI)^(5)^ em sua versão adaptada para o português do Brasil^(37)^. O tempo de referência para responder aos itens do instrumento era o último mês, o que possibilitou a avaliação do autocuidado do participante no período que antecedeu sua hospitalização.

O instrumento contém 22 questões, divididas em três subescalas: Manutenção do autocuidado (10 itens); Manejo do autocuidado (6 itens) e Confiança no autocuidado (6 itens). Na subescala Manutenção, as respostas para cada item variam de nunca/raramente (valor 1) a sempre/diariamente (valor 4), exceto para o item 8, cujos valores são reversos; na subescala Manejo, variam de pouco provável (valor 1) a muito provável (valor 4) e, na subescala Confiança, de não confiante (valor 1) a extremamente confiante (valor 4). As somas dos valores respondidos para cada subescala, posteriormente, são transformadas em uma escala entre zero e 100, de modo que valores maiores indicam melhor autocuidado na respectiva subescala. Os resultados também podem ser interpretados de forma categorizada: valores acima de 70 pontos indicam autocuidado adequado, enquanto valores iguais ou menores que 70 informam autocuidado inadequado^(5)^.

A escolha do SCHFI foi baseada nos resultados de validade e confiabilidade verificados na versão original^(5)^ e na versão adaptada para o Brasil^(37)^. A validade de construto da versão brasileira foi demonstrada pelos resultados dos testes de correlação entre as suas medidas de autocuidado com aquelas advindas de questões equivalentes da versão brasileira da *European Heart Failure Self-care Behavior Scale* (r = −0,51; p < 0,001) e pela confirmação das três subescalas da versão original, conforme a análise fatorial confirmatória. A presença de adequada consistência interna de seus itens foi verificada pelos valores obtidos do alfa de Cronbach, de 0,40; 0,82 e 0,93, respectivamente, para as subescalas Manutenção, Manejo e Confiança^(37)^. A versão brasileira foi utilizada em outros estudos antes da ocorrência da pandemia^(10,12)^.

Para avaliação da fadiga, utilizamos o Pictograma de Fadiga^(38)^, na versão validada para o Brasil^(39)^, a qual é composta por duas questões. A primeira questão é: “Quanto cansado você se sentiu na última semana?”, com as seguintes opções de resposta: nada cansado; um pouquinho cansado; moderadamente cansado; muito cansado e extremamente cansado. A segunda é: “O quanto a sensação de cansaço impede você de fazer o que quer fazer?”, e as respostas possíveis são: eu consigo fazer tudo que habitualmente faço; eu consigo fazer quase tudo que habitualmente faço; eu consigo fazer algumas das coisas que habitualmente faço; eu só faço o que tenho que fazer e eu consigo fazer muito pouco. As respostas são acompanhadas por dois conjuntos de figuras para ajudar na avaliação da intensidade (questão 1) e do impacto (questão 2) da fadiga. A versão brasileira foi considerada como uma medida válida e confiável para pacientes oncológicos^(39)^. Posteriormente, estudo realizado com pacientes com IC constatou associação entre as classes funcionais da NYHA com os valores das questões do instrumento, mostrando sua validade para analisar a fadiga também nesse grupo^(32)^.

Como o tempo de referência para as respostas dos itens dos instrumentos de autocuidado e fadiga são, respectivamente, o último mês e a última semana, orientávamos que os participantes considerassem tais períodos antes da internação atual.

### Análise dos resultados e estatística

Os dados foram processados e analisados descritivamente pelo programa *Statistical Package for the Social Science*, da *International Business Machines Corporation*™ (IBM™ - SPSS), na versão 24.1 para *Windows*.

Para testar a associação entre o autocuidado e a fadiga, utilizamos o teste de Kruskal-Wallis. O nível de significância adotado foi de 0,05.

### Aspectos éticos

O estudo foi conduzido de acordo com as diretrizes de ética nacionais e internacionais e aprovado pelo Comitê de Ética em Pesquisa da Escola de Enfermagem de Ribeirão Preto (CAAE nº 25497912.2.0000.5393). O consentimento livre e esclarecido foi obtido de todos os indivíduos envolvidos no estudo por meio de termo escrito.

## Resultados

Durante o período do estudo, recrutamos 137 potenciais participantes. Desses, cinco foram excluídos por não apresentarem orientação alopsíquica. Os 132 participantes apresentaram média de idade de 62,3 e desvio-padrão de 28,7 anos. A maioria era do sexo masculino (55,3%), casados/em união estável (58,3%), com escolaridade de até nove anos de estudo (74,2%), provenientes de outras cidades do estado de São Paulo que não Ribeirão Preto (65,9%), residiam com cônjuge e/ou filhos (58,4%). Em relação às características clínicas, 59,1% tinham FEVE reduzida e encontravam-se na classe funcional IV, segundo a NYHA (56,8%) (Tabela 1).

Na medida de autocuidado da IC, verificamos que os escores médios ficaram abaixo da pontuação esperada para o autocuidado adequado (acima de 70) nas subescalas Manutenção e Manejo. Na subescala Manutenção, o escore médio foi de 54,1, enquanto na subescala Manejo foi de 60,6. Por sua vez, a subescala Confiança atingiu valor limítrofe de 70. Ao verificarmos a distribuição dos participantes segundo a realização do autocuidado adequado, constatamos as seguintes frequências: 17,4% dos participantes na subescala Manutenção, 38,2% na subescala Manejo e 51,7% na subescala Confiança (Tabela 2).

**Tabela 1-:** Caracterização sociodemográfica e clínica dos 132 participantes. Ribeirão Preto, SP, Brasil, 2021-2022

**Características sociodemográficas e clínicas**	n (%)*	**Média (D.P.)** ^†^
**Idade (anos)**		62,3 (28,7)
**Sexo**		
Masculino	73 (55,3)	
Feminino	59 (44,7)	
**Situação conjugal**		
Casado/união estável	77 (58,3)	
Solteiro	24 (18,2)	
Viúvo	18 (13,6)	
Desquitado/divorciado	13 (9,8)	
**Escolaridade (anos que frequentou a escola)**		5,9 (4,2)
Até 9	98 (74,2)	
10-12 anos	25 (18,9)	
13 ou mais	9 (6,8)	
**Renda familiar mensal (em reais)**		2.397,88 (1.285,5)
**Cidade em que reside**		
Ribeirão Preto	41 (31,1)	
Outras cidades do estado de São Paulo	87 (65,9)	
**Classe funcional NYHA** ^‡^ **(autorreferida)**		
Classe 1	9 (6,8)	
Classe 2	16 (12,1)	
Classe 3	23 (17,4)	
Classe 4	75 (56,8)	
**FEVE** ^§^		
Preservada	28 (21,2)	
Intermediária	12 (9,1)	
Reduzida	78 (59,1)	
Sem informação	14 (10,6)	

*n (%) = Número (porcentagem); ^†^D.P. = Desvio-padrão; ^‡^NYHA = *New York Heart Association*; ^§^FEVE = Fração de ejeção do ventrículo esquerdo

**Tabela 2-:** Avaliação do autocuidado dos 132 participantes, segundo o *Heart Failure Index*. Ribeirão Preto, SP, Brasil, 2021-2022

Medidas subescalas SCHFI*	**n (%)** ^†^	**Média (D.P.)** ^‡^	**Mediana (mínimo-máximo)**
**Manutenção (n=132)**		54,1 (18,0)	53,3 (3,3 – 96,6)
Adequado	23 (17,4)		
Inadequado	109 (82,6)		
**Manejo (n=131)**		60,6 (20,0)	65 (0 – 100)
Adequado	50 (38,2)		
Inadequado	81 (61,8)		
**Confiança (n=118)**		70 (19,1)	72,3 (22,2 – 100)
Adequado	61 (51,7)		
Inadequado	57 (48,3)		

*SCHFI = *Self-care Heart Failure Index*; ^†^n (%) = Número (porcentagem); ^‡^D.P. = Desvio-padrão

Na subescala Manutenção, as ações de autocuidado mais realizadas foram: “ser assíduo na consulta com médico ou enfermeiro” (84,8%), “esquece-se ou deixa de tomar algum de seus medicamentos” (valores reversos) (68,8%), “tenta evitar ficar doente (por exemplo, vacinar-se contra a gripe, evitar contato com pessoas doentes)” (74,1%), “ingere uma dieta com pouco sal” (56,3%) e “verifica se os tornozelos estão inchados” (48,2%). Em contrapartida, as ações “exercita-se por 30 minutos” (75,9%), “realiza alguma atividade física” (66,1%) e “solicita alimentos com pouco sal quando come fora ou visita alguém” (61,6%) foram respondidas com as alternativas nunca ou raramente pelos participantes.

Na subescala Manejo, as ações “contatar seu médico ou enfermeiro para orientação” (47,7%) e “reduzir o sal na dieta” (46,2%) foram consideradas de realização muito provável se os pacientes apresentassem dificuldade para respirar ou inchaço nos tornozelos. A maioria (90,2%) teve problemas para respirar ou tornozelos inchados no último mês, sendo que 18,8% não reconheceram tais sintomas como sendo da IC; 29,9% reconheceram rapidamente e 26,8% reconheceram imediatamente. A partir dos recursos presentes nos itens dois, três, quatro e cinco da subescala Manejo, 28,6% dos participantes responderam que não tentaram algum dos recursos citados da última vez que tiveram dificuldades para respirar ou inchaço nos tornozelos. Dentre os que tentaram algum dos recursos, 27,7% referiram ter absoluta certeza de que o recurso os ajudou, 23,2% referiram ter certeza, 8,9% pouca certeza e 9,8% não ter certeza.

Para a subescala Confiança, os itens com maior confiança foram: “seguir o tratamento recomendado” (45,5%), “avaliar a importância de seus sintomas” (42,0%) e “avaliar se um medicamento funciona” (37,5%). O item com menor confiança foi “estar livre dos sintomas de IC” (23,2%).

A aplicação do Pictograma de Fadiga evidenciou que a maioria dos participantes se sentia muito cansada (39,4%) ou extremamente cansada (29,5%). A sensação de cansaço interferia na execução de suas atividades de vida diária e, consequentemente, na qualidade de vida, visto que 49% alegaram que, devido à sensação incapacitante, conseguiam fazer muito pouco ou apenas o que tinham que fazer (20,5%) (Tabela 3).

**Tabela 3-:** Avaliação da fadiga dos 132 participantes, segundo o Pictograma de Fadiga. Ribeirão Preto, SP, Brasil, 2021-2022

**Pictograma de Fadiga**	n (%)*
**Item 1 – Quanto cansado você se sentiu na última semana?**	
Nada cansado	9 (6,8)
Um pouquinho cansado	12 (9,1)
Moderadamente cansado	20 (15,2)
Muito cansado	52 (39,4)
Extremamente cansado	39 (29,5)
**Item 2 – O quanto a sensação de cansaço te impede você de fazer o que quer fazer?**	
Eu consigo fazer tudo que habitualmente faço	7 (5,3)
Eu consigo fazer quase tudo que habitualmente faço	9 (6,8)
Eu consigo fazer algumas das coisas que habitualmente faço	24 (18,2)
Eu só faço o que tenho que fazer	27 (20,5)
Eu consigo fazer muito pouco	65 (49,2)

*n (%) = Número (porcentagem)

A possível associação entre as medidas de autocuidado e de fadiga foi avaliada e não constatamos resultados estatisticamente significantes entre a intensidade da fadiga e as subescalas Manutenção (p = 0,266), Manejo (p = 0,074) e Confiança (p = 0,059) para o autocuidado. Resultados semelhantes foram observados entre o impacto da fadiga e as subescalas Manutenção (p = 0,834), Manejo (p = 0,609) e Confiança (p = 0,139) para o autocuidado dos participantes.

As distribuições das respostas obtidas nas subescalas de Manutenção, Manejo e Confiança do autocuidado dos participantes, segundo a intensidade (questão 1) e o impacto (questão 2) da fadiga, estão apresentadas, respectivamente, nas Figuras 1 e 2.

**Figura 1-:** *Boxplots*com os resultados das medidas de Manutenção, Manejo e Confiança para o autocuidado da insuficiência cardíaca, segundo a intensidade da fadiga

**Figura 2-:** *Boxplots* com os resultados das medidas de Manutenção, Manejo e Confiança para o autocuidado da insuficiência cardíaca, segundo o impacto da fadiga

## Discussão

O estudo objetivou verificar a associação entre o autocuidado e a fadiga de pacientes internados por descompensação da IC durante a pandemia da COVID-19. Constatamos manutenção e manejo inadequados do autocuidado no mês que antecedeu a hospitalização pela descompensação clínica da IC. Na semana que antecedeu a internação, a maioria dos participantes relatou fadiga intensa e comprometimento das atividades diárias. Entretanto, não constatamos associações estatisticamente significantes entre as subescalas Manutenção, Manejo e Confiança para o autocuidado da IC e a intensidade da fadiga e seu impacto nas atividades diárias realizadas.

Por se tratar de um estudo observacional transversal, que investigou uma amostra não probabilística de 132 pacientes, consideramos os resultados como sendo de natureza exploratória. Os resultados obtidos não podem ser generalizados para todos os pacientes com as mesmas características clínicas que foram hospitalizados no Brasil durante a pandemia da COVID-19. Trata-se de uma amostra representativa, é dizer, cujos participantes, embora não tenham sido escolhidos de forma aleatória, representam a população a ser estudada. Os resultados obtidos, assim como os de outros estudos que utilizaram amostras de populações clínicas^(12,32-33)^ trazem contribuições importantes para o planejamento de estudos de intervenção^(34-35)^, pois permitirão a determinação do cálculo amostral e do tamanho da amostra desses estudos.

A interpretação dos resultados obtidos deve ser considerada tendo em vista o contexto epidemiológico no qual a pesquisa foi realizada, bem como o fato de os participantes terem sido recrutados de um único serviço de saúde (hospital público e de ensino do interior do estado de São Paulo), as características demográficas que caracterizam os pacientes atendidos pelo Sistema Único de Saúde e, principalmente, a utilização dos instrumentos de medidas escolhidos dentre uma gama de outras opções. O delineamento transversal do estudo não permite inferir a associação de causa e efeito entre fadiga e autocuidado na amostra estudada. A declaração da Organização Mundial da Saúde sobre o fim do status de emergência global da pandemia da COVID-19 impossibilitará a realização de estudos de acompanhamento da população de interesse, cujo delineamento prospectivo poderia investigar a relação das duas variáveis com a descompensação clínica da IC.

Durante a pandemia da COVID-19, as ações de autocuidado para pessoas com IC diminuíram^(27-30)^, havendo dificuldades para o acesso aos serviços de saúde, interferindo no acompanhamento presencial dos pacientes. No Brasil, a baixa adesão ao tratamento farmacológico e dietético foram os principais fatores desencadeantes de IC descompensada durante a pandemia, entre março e outubro de 2020^(40)^. Com a limitação das consultas e o medo do contágio^(20,25)^, os pacientes com IC reduziram a busca pelos serviços de saúde no Brasil^(41)^ e em outros países^(20,23,25,42-44)^, o que contribuiu para o aumento da mortalidade^(44)^.

O perfil sociodemográfico dos participantes, com a predominância do sexo masculino, idosos, casados ou em união estável, que não realizam trabalho remunerado, de baixa renda e pouca escolaridade, também foi observado em outros estudos nacionais e internacionais^(30,32,41,45)^.

Os resultados obtidos também devem ser interpretados levando em consideração os instrumentos de medidas escolhidos para a avaliação do autocuidado com a IC^(5,37)^ e a fadiga de indivíduos com esse diagnóstico^(38-39)^. As duas escalas possibilitaram a comparação dos resultados obtidos no período pandêmico com outras investigações realizadas antes e durante a pandemia da COVID-19.

Ao compararmos os resultados obtidos nas subescalas da SCHFI com aqueles encontrados por pesquisadores brasileiros em estudos anteriores à pandemia, constatamos valores semelhantes entre pacientes atendidos no ambulatório do hospital onde realizamos o nosso estudo, os quais foram acompanhados nos três primeiros meses após a alta da internação por descompensação da IC^(12)^. Em outro estudo, realizado em hospital de ensino da cidade de São Paulo, com pacientes ambulatoriais, valores semelhantes foram reportados pelos pesquisadores^(10)^. Os nossos resultados foram semelhantes aos observados por pesquisadores que usaram a mesma ferramenta para avaliar o autocuidado de pacientes nos Estados Unidos da América^(27,29)^, Suíça^(18)^ e China^(45)^.

Com relação ao sintoma de fadiga, no grupo estudado, a intensidade da fadiga (muito ou extremamente cansado) e sua interferência na realização das atividades diárias foram reportadas pelos participantes. A presença do sintoma fadiga em pessoas com IC encontra-se bem documentada^(32,46)^, interferindo na realização de atividades físicas^(6)^, no autocuidado^(6-8,47)^ e na qualidade de vida dos pacientes^(5,8)^. Durante a pandemia, houve a redução da atividade física e o aumento das manifestações de sintomas de descompensação da IC, como a fadiga^(26)^. Indivíduos que apresentavam melhor controle dos sintomas antes da pandemia mantiveram o melhor autocuidado durante a pandemia quando comparados com aqueles com autocuidado deficiente^(28)^. Embora tenhamos encontrado poucos estudos que avaliaram a fadiga com o mesmo instrumento^(6,32)^, nossos resultados corroboram aqueles constatados em pacientes com IC antes da pandemia.

Os resultados obtidos corroboram aqueles reportados por outros pesquisadores e reforçam a necessidade de intervenções de enfermagem voltadas à literacia em saúde para o autocuidado desses indivíduos. Estudos apontam que a telemedicina teve papel importante durante o período pandêmico, permitindo a monitorização, identificação dos pacientes prioritários e a realização de orientações em saúde^(41,48-51)^. Essa modalidade de atendimento já era empregada antes da pandemia, entretanto não havia consenso com relação à sua eficácia nos pacientes com IC^(52)^. A pandemia foi um fator que impulsionou sua implementação em maior escala, de modo que ela se mostrou uma alternativa eficiente^(26,28,52)^.

A enfermagem deve considerar o autocuidado na IC como um processo ativo, a ser planejado em conjunto com o indivíduo e seus familiares, envolvendo desde o reconhecimento de sintomas de descompensação clínica até a tomada de decisão para procurar por atendimento de saúde. Assim, a educação em saúde visa obter resultados positivos de adesão aos tratamentos farmacológico e não farmacológico, favorecendo o manejo, a manutenção e a confiança para o autocuidado das pessoas com IC.

Quanto à contribuição do estudo para o avanço do conhecimento científico da presente temática, reforçamos a existência de autocuidado insatisfatório da IC nas quatro semanas que antecederam a necessidade de internações não planejadas decorrentes da descompensação clínica da doença. Ao compararmos os resultados do presente estudo com aqueles provenientes de estudos anteriores, observamos que, independentemente da existência da pandemia da COVID-19, pessoas com IC ainda necessitam de educação voltada ao autocuidado da sua condição de saúde, sendo o enfermeiro um dos profissionais mais qualificados para planejar essa complexa intervenção, não apenas para os pacientes, mas também para seus familiares/cuidadores.

As principais limitações do estudo foram: a impossibilidade de cálculo amostral com a determinação do número necessário de participantes para garantir o caráter confirmatório do resultado obtido; o delineamento transversal, o qual não permite ao pesquisador inferência sobre a causalidade das variáveis, no caso, autocuidado e fadiga e o fato de o recrutamento dos participantes ter ocorrido em uma única instituição hospitalar.

## Conclusão

Durante a pandemia da COVID-19, pessoas internadas pela descompensação clínica da IC avaliaram o autocuidado nas quatro semanas que antecederam a hospitalização como sendo inadequado para manejo, manutenção e conhecimento da IC. A fadiga foi considerada pela maioria como intensa na semana que antecedeu a internação, comprometendo o desenvolvimento das atividades diárias. Ao verificarmos a presença de possível associação entre o autocuidado e a fadiga, não constatamos resultados estatisticamente significantes no grupo investigado.

## Data Availability

Os conjuntos de dados relacionados a este artigo estarão disponíveis mediante solicitação ao autor correspondente.

# Translation: Autocuidado y fatiga en individuos hospitalizados con insuficiencia cardíaca descompensada durante la pandemia de COVID-19: un estudio transversal

**DOI:** 10.1590/1518-8345.7465.4616

**Authors:** Laura da Silva Araujo, Kethlen Louise Palha Ferrari, Pedro Paulo Fernandes de Aguiar Tonetto, Daiane Vieira Medeiros Costa Zanetti, Carina Aparecida Marosti Dessotte, Rosana Aparecida Spadoti Dantas

**Affiliations:** 1 Universidade de São Paulo, Escola de Enfermagem de Ribeirão Preto, Centro Colaborador de la OPS/OMS para el Desarrollo de la Investigación en Enfermería, Ribeirão Preto, SP, Brasil. Universidade de São Paulo Escola de Enfermagem de Ribeirão Preto Centro Colaborador de la OPS/OMS para el Desarrollo de la Investigación en Enfermería SP Ribeirão Preto Brasil; 2 Becaria del Conselho Nacional de Desenvolvimento Científico e Tecnológico (CNPq), Brasil. Becaria del Conselho Nacional de Desenvolvimento Científico e Tecnológico Brasil; 3 Becario de la Coordenação de Aperfeiçoamento de Pessoal de Nível Superior (CAPES), Brasil. Becario de la Coordenação de Aperfeiçoamento de Pessoal de Nível Superior Brasil

**Keywords:** Autocuidado, Fatiga, Insuficiencia Cardíaca, COVID-19, Enfermería, Cardiología

## Abstract

verificar la asociación entre el autocuidado y la fatiga en pacientes hospitalizados por insuficiencia cardíaca descompensada durante la pandemia de COVID-19.

estudio observacional, de corte transversal, con 132 individuos ingresados en un hospital universitario. Los datos se recolectaron mediante entrevistas individuales y consulta de historias clínicas. El autocuidado se evaluó mediante el *Self-Care of Heart Failure Index* y la fatiga mediante el Pictograma de Fatiga, ambas versiones validadas previamente para su uso en Brasil. Valores iguales o mayores a 70 indican un adecuado autocuidado para Manejo, Mantenimiento y Confianza.

la mayoría de los participantes era hombre (n = 73; 55,3%), con baja escolaridad (n = 98; 74,2%), vivía con cónyuge o hijos (n = 77; 58,4%) y la media de edad fue de 62,3 años (desviación estándar = 28,7). Constatamos un autocuidado inadecuado de la insuficiencia cardíaca en las subescalas Mantenimiento (n = 109; 82,6%), Manejo (n = 81; 61,8%) y Confianza (n = 57; 48,3%). Considerando la última semana antes de la hospitalización, los participantes refirieron cansancio y limitaciones producto de la fatiga. No constatamos asociación estadísticamente significativa entre la intensidad y el impacto de la fatiga y las subescalas de autocuidado.

durante el período pandémico, la fatiga percibida por los participantes antes de la hospitalización por la descompensación clínica de la insuficiencia cardíaca, aunque prevalente, no tuvo asociación con el autocuidado de la enfermedad.

## Destacados

**(1)** El autocuidado de la insuficiencia cardíaca fue inadecuado durante la pandemia. **(2)** La fadiga fue un síntoma prevalente e incapacitante antes de la hospitalización. **(3)** Los autocuidados de la insuficiencia cardíaca deben ser priorizados por el personal de enfermaría.

## Introducción

La insuficiencia cardíaca (IC) es un síndrome multifactorial crónico causado por alteraciones estructurales o funcionales^(1)^ que comprometen la capacidad del corazón para bombear sangre para satisfacer las necesidades metabólicas tisulares, lo que resulta en una reducción del débito cardíaco^(2)^. La manifestación súbita o gradual de signos y síntomas de IC como disnea, disnea paroxística nocturna, ortopnea, arritmias, edema de miembros inferiores y fatiga, caracterizan la descompensación de la enfermedad^(3)^. El autocuidado orientado al manejo de la IC ha demostrado ser esencial para prevenir la descompensación de la enfermedad, que es el objetivo de la investigación del estudio. Adoptamos la definición de autocuidado, propuesta por Bárbara Riegel y colaboradores, como resultado de un proceso de toma de decisiones que implica acciones de mantenimiento, manejo y confianza para el autocuidado en personas con IC^(4-5)^. El síntoma fatiga se ha asociado con barreras a la adhesión a conductas adecuadas de autocuidado al promover dificultades físicas y emocionales, lo que compromete la capacidad del individuo para cuidar de sí mismo^(6-8)^.

El autocuidado es un factor fundamental para la adhesión a un estilo de vida saludable y el manejo de los signos y síntomas^(9)^. En el caso de la IC, contribuye al control de la enfermedad, mejora la calidad de vida, reduce el número de hospitalizaciones por descompensación clínica y los costos de los servicios de salud^(10-13)^.

La relevancia de la investigación sobre autocuidado se basa en estudios de revisión actuales sobre la necesidad de intervenciones dirigidas a promover el autocuidado en personas con IC^(11,14)^. En el contexto de la práctica clínica, la enfermería desempeña un papel fundamental en la educación para la salud. En la literatura consta el uso aislado o combinado de diferentes estrategias de enseñanza, siendo las más evidentes: orientación personalizada basada en la exploración ecográfica de la vena cava inferior, consultas telefónicas, aplicación de folletos y conformación de un grupo educativo^(10,15)^. Sin embargo, es necesario realizar mayores inversiones para mejorar las conductas de autocuidado, reducir el número de reingresos hospitalarios no planificados y mantener la calidad de vida de estos individuos^(15-19)^. El conocimiento de los pacientes sobre la enfermedad contribuye a su comprensión de las pautas de salud y a su seguimiento clínico en los diferentes niveles de cuidado^(19)^.

Durante la trayectoria de la IC, se pueden requerir regulaciones y adaptaciones en la planificación del autocuidado, como, por ejemplo, cuando hay un período de empeoramiento de la enfermedad, desarrollo de nuevas comorbilidades o situaciones que requieren terapias más avanzadas. Durante la pandemia de coronavirus (COVID-19), se constató el empeoramiento de las condiciones de salud de las personas con diagnóstico de IC, con mayor agravamiento del cuadro clínico ya existente^(20)^, empeoramiento del pronóstico y un mayor riesgo de mortalidad entre aquellos infectados por el SARS-CoV-2^(21-24)^. Las acciones de autocuidado adquirieron mayor importancia durante el período pandémico, con el fin de evitar hospitalizaciones por descompensación de la enfermedad cardíaca, así como el contagio con el nuevo coronavirus^(25)^.

En otros países, como Francia^(26)^ y Estados Unidos de América^(27-29)^ se realizaron estudios para evaluar el autocuidado de las personas con IC durante la pandemia. En Brasil, se identificó un solo estudio hasta el momento^(30)^. Los autores investigaron a pacientes que habían sido hospitalizados debido a una descompensación clínica y concluyeron que la falta de seguimiento por parte de los profesionales de la salud impactó en el manejo del autocuidado de dichos pacientes^(30)^. El presente estudio tuvo como objetivo verificar la asociación entre el autocuidado y la fatiga de pacientes hospitalizados por descompensación de IC durante la pandemia de COVID-19.

## Método

### Diseño del estudio

Estudio observacional, de corte transversal^(31)^.

El artículo fue reportado según los *Guidelines Strengthening the Reporting of Observational Studies in Epidemiology* (STROBE), recomendados por la *TheEnhancing the Quality and Transparency of Health ResearchNetwork* (EQUATOR).

### Lugar de estudio

El estudio se realizó en las unidades de internación de la división de cardiología del Hospital de Clínicas de la Facultad de Medicina de Ribeirão Preto de la *Universidade de São Paulo*, ubicado en el interior del estado de São Paulo, Brasil. Se trata de un hospital público y universitario, de nivel terciario, vinculado a la Facultad de Medicina de Ribeirão Preto de la Universidad de São Paulo. Atiende a la población de Ribeirão Preto y de otras ciudades del estado de São Paulo, así como de otros estados brasileños y es el hospital público más grande de la región.

### Población accesible y criterios de elegibilidad de la muestra

La población accesible estuvo constituida por individuos ingresados en el mencionado hospital durante la pandemia de COVID-19 con diagnóstico de descompensación clínica de IC. Cabe resaltar que las investigaciones en Enfermería han constatado la necesidad de que ocurra un evento clínico para la elegibilidad de los participantes en estudios observacionales^(12,32-33)^ y de intervención^(34-35)^. Por lo tanto, el tamaño final de la muestra se basó en la incidencia del evento clínico, es decir, la hospitalización de pacientes por descompensación clínica, durante los meses de mayo del 2021 a octubre del 2022. Se trata, por lo tanto, de un subconjunto de la población objetivo que se ha definido geográfica y temporalmente^(31)^.

Investigamos una muestra consecutiva no probabilística, conformada por aquellos individuos que cumplieron con los criterios de elegibilidad: tener 18 años de edad o más; estar ingresado en la institución hospitalaria mencionada durante el período de mayo del 2021 a octubre del 2022; estar clínicamente estable, sin presencia de fatiga o disnea en el momento de la entrevista^(12,32,34)^.

Para evaluar la orientación alopsíquica, a los participantes se les hicieron seis preguntas, a saber: su nombre, fecha actual, día de la semana, ubicación, edad y ciudad de residencia. Se excluyeron aquellos que respondieron de forma incorrecta más de dos preguntas^(32,35-36)^.

### Recopilación de datos

Para identificar a los participantes potenciales, se realizó una búsqueda activa y regular en las salas de enfermería de cardiología durante todo el período del estudio. La regularidad de la búsqueda permitió la identificación de todos los pacientes hospitalizados durante la investigación. Los datos se obtuvieron mediante entrevistas individuales y consultas a las historias clínicas electrónicas de los participantes. Se recopiló información sociodemográfica, específicamente, sexo, edad, ingresos familiares mensuales, estado civil, ciudad de residencia, educación (años de educación formal) y situación profesional; y clínica, específicamente, medicamentos utilizados, comorbilidades, etiología de la IC, clase funcional autoinformada, adaptada de *New York Heart Association* (NYHA) y la fracción de eyección del ventrículo izquierdo (FEVI), obtenida del examen ecocardiográfico, posteriormente, categorizada como conservada (>50%), intermedia (40-49%) y reducida (≤40%)]^(3)^.

El autocuidado en la IC se evaluó mediante el *Self-Care of Heart Failure Index* (SCHFI)^(5)^ en su versión adaptada al portugués de Brasil^(37)^. El tiempo de referencia para responder las preguntas del instrumento fue el último mes, lo que permitió evaluar el autocuidado del participante en el período previo a su hospitalización.

El instrumento contiene 22 preguntas divididas en tres subescalas: Mantenimiento del autocuidado (10 preguntas), Manejo del autocuidado (6 preguntas) y Confianza en el autocuidado (6 preguntas). En la subescala Mantenimiento, las respuestas para cada pregunta varían de nunca/rara vez (valor 1) a siempre/diariamente (valor 4), salvo para la pregunta 8, cuyos valores están invertidos; en la subescala Manejo, varían de poco probable (valor 1) a muy probable (valor 4); y, en la subescala Confianza, de nada seguro (valor 1) a extremadamente seguro (valor 4). Las sumas de los valores respondidos para cada subescala se transforman, posteriormente, en una escala entre cero y 100, indicando valores más altos un mejor autocuidado en la respectiva subescala. Los resultados también pueden interpretarse de forma categorizada: valores superiores a 70 puntos indican un autocuidado adecuado, mientras que valores iguales o inferiores a 70 indican un autocuidado inadecuado^(5)^.

La elección del SCHFI se basó en los resultados de validez y confiabilidad verificados en la versión original^(5)^ y en la versión adaptada para Brasil^(37)^. La validez de constructo de la versión brasileña fue demostrada por los resultados de las pruebas de correlación entre sus medidas de autocuidado y las que surgieron de preguntas equivalentes en la versión brasileña de la *European Heart Failure Self-care Behavior Scale* (r = − 0,51; p < 0,001) y por la confirmación de las tres subescalas de la versión original, según el análisis factorial confirmatorio. La presencia de una adecuada consistencia interna de sus preguntas se comprobó por los valores obtenidos del alfa de Cronbach de 0,40; 0,82 y 0,93, respectivamente para las subescalas de Mantenimiento, Manejo y Confianza^(37)^. La versión brasileña se utilizó en otros estudios antes de que ocurriera la pandemia^(10,12)^.

Para evaluar la fatiga utilizamos el Pictograma de Fatiga^(38)^, en la versión validada para Brasil^(39)^, que consta de dos preguntas. La primera pregunta es “¿En qué medida te has sentido cansado en la última semana?” con las siguientes opciones de respuesta: nada cansado; un poco cansado; moderadamente cansado; muy cansado y sumamente cansado. La segunda es: “¿En qué medida la sensación de cansancio te impide hacer lo que quieres hacer?” y las posibles respuestas son: puedo hacer todo lo que hago habitualmente; puedo hacer casi todo lo que hago habitualmente; puedo hacer algunas de las cosas que hago habitualmente; simplemente hago lo que necesito hacer y puedo hacer muy poco. Las respuestas van acompañadas de dos conjuntos de figuras para ayudar a evaluar la intensidad (pregunta 1) y el impacto (pregunta 2) de la fatiga. La versión brasileña se consideró una medida válida y confiable para pacientes oncológicos^(39)^. Posteriormente, un estudio realizado con pacientes con IC constató una asociación entre las clases funcionales de la NYHA con los valores de las preguntas del instrumento demostrando su validez para analizar la fatiga también en este grupo^(32)^.

Como el tiempo de referencia para las respuestas a las preguntas de los instrumentos de autocuidado y fatiga son, respectivamente, el último mes y la última semana, aconsejamos a los participantes considerar estos períodos anteriores a la hospitalización actual.

### Análisis de resultados y estadísticas

Los datos fueron procesados y analizados descriptivamente por el programa *Statistical Package for the Social Science,* de la *International Business Machines Corporation*™ (IBM™ - SPSS), versión 24.1 para *Windows*.

Para probar la asociación entre el autocuidado y la fatiga, utilizamos la prueba de Kruskal-Wallis. El nivel de significación adoptado fue de 0,05.

### Aspectos éticos

El estudio se realizó según las directrices éticas nacionales e internacionales y fue aprobado por el Comité de Ética en Investigación de la Escuela de Enfermería de Ribeirão Preto (CAAE n.º 25497912.2.0000.5393). Se obtuvo el consentimiento libre, previo e informado de todas las personas que participaron en el estudio mediante un formulario escrito.

## Resultados

Durante el período de estudio reclutamos 137 participantes potenciales. De estos, cinco fueron excluidos por no presentar orientación alopsíquica. Los 132 participantes tenían una edad media de 62,3 y una desviación estándar (D.E.) de 28,7 años. La mayoría eran hombres (55,3%), casados/en unión estable (58,3%), con hasta nueve años de escolaridad (74,2%), originarios de otras ciudades del estado de São Paulo (65,9%), que vivían con cónyuge o hijos (58,4%). En cuanto a las características clínicas, el 59,1% presentó FEVI reducida y se encontraba en clase funcional IV, según la NYHA (56,8%) (Tabla 1).

En la medida de autocuidado de la IC, encontramos que los puntajes promedio estaban por debajo del puntaje esperado para un autocuidado adecuado (por encima de 70) en las subescalas “Mantenimiento” y “Manejo”. En la subescala Mantenimiento, el puntaje promedio fue de 54,1, mientras que en la subescala Manejo fue de 60,6. A su vez, la subescala Confianza alcanzó un valor límite de 70. Al verificar la distribución de los participantes según el desempeño del autocuidado adecuado, encontramos las siguientes frecuencias: El 17,4% de los participantes en la subescala “Mantenimiento”, el 38,2% en la subescala “Manejo” y el 51,7% en la subescala “Confianza” (Tabla 2).

**Tabla 1-:** Caracterización sociodemográfica y clínica de los 132 participantes. Ribeirão Preto, SP, Brasil, 2021-2022

**Características sociodemográficas y clínicas**	n (%)*	**Media (D.E.)** ^†^
**Edad (en años)**		62,3 (28,7)
**Sexo**		
Masculino	73 (55,3)	
Femenino	59 (44,7)	
**Estado civil**		
Casado/unión estable	77 (58,3)	
Soltero	24 (18,2)	
Viudo	18 (13,6)	
Separado/divorciado	13 (9,8)	
**Escolaridad (años de asistencia a la escuela)**		5,9 (4,2)
Hasta 9	98 (74,2)	
10 - 12 años	25 (18,9)	
13 o más	9 (6,8)	
**Ingresos familiares mensuales (en reales)**		2.397,88 (1.285,5)
**Ciudad donde vive**		
Otras ciudades del estado de São Paulo	87 (65,9)	
Ribeirão Preto	41 (31,1)	
**Clase funcional NYHA** ^‡^ **(autoinformada)**		
Clase 1	9 (6,8)	
Clase 2	16 (12,1)	
Clase 3	23 (17,4)	
Clase 4	75 (56,8)	
**FEVI** ^§^		
Conservada	28 (21,2)	
Intermedia	12 (9,1)	
Reducida	78 (59,1)	
Sin información	14 (10,6)	

*n (%) = Número (porcentaje); ^†^D.E. = Desviación estándar; ^‡^NYHA = *New York Heart Association*; ^§^FEVI = Fracción de Eyección del Ventrículo Izquierdo

**Tabla 2-:** Evaluación del autocuidado de los 132 participantes, según el *Self-Care Heart Failure Index*. Ribeirão Preto, SP, Brasil, 2021-2022

Medidas subescalas SCHFI*	**n (%)** ^†^	**Media (D.E.)** ^‡^	**Mediana (mínimo-máximo)**
**Mantenimiento (n=132)**		54,1 (18,0)	53,3 (3,3 – 96,6)
Adecuado	23 (17,4)		
Inadecuado	109 (82,6)		
**Manejo (n=131)**		60,6 (20,0)	65 (0 – 100)
Adecuado	50 (38,2)		
Inadecuado	81 (61,8)		
**Confianza (n=118)**		70 (19,1)	72,3 (22,2 – 100)
Adecuado	61 (51,7)		
Inadecuado	57 (48,3)		

*SCHFI = *Self-Care Heart Failure Index*; ^†^n (%) = Número (porcentaje); ^‡^D.E. = Desviación estándar

En la subescala Mantenimiento, las acciones de autocuidado más realizadas fueron las siguientes: “ser asiduo a las citas con el médico o enfermero” (84,8%), “olvidar o dejar de tomar alguno de sus medicamentos” (valores inversos) (68,8%), “tratar de evitar enfermarse (por ejemplo: vacunarse contra la gripe, evitar el contacto con personas enfermas)” (74,1%), “llevar una dieta baja en sal” (56,3%) y “revisar si tiene los tobillos hinchados” (48,2%). Mientras que las acciones “hacer ejercicio durante 30 minutos” (75,9%), “realizar alguna actividad física” (66,1%) y “pedir comida baja en sal al comer fuera o al visitar a alguien” (61,6%) fueron respondidas con las alternativas nunca o rara vez por los participantes.

En la subescala Manejo, las acciones “contactar a su médico o enfermero para obtener orientación” (47,7%) y “reducir la sal en su dieta” (46,2%) se consideraron muy probables de realizar si los pacientes tenían dificultad para respirar o hinchazón en los tobillos. La mayoría (90,2%) tuvo problemas para respirar o tobillos hinchados en el último mes, y el 18,8% no reconoció estos síntomas como relacionados con la IC; el 29,9% lo reconoció rápidamente y el 26,8% lo reconoció inmediatamente. Con base en los recursos presentes en las preguntas dos, tres, cuatro y cinco de la subescala Manejo, el 28,6% de los participantes respondió que no probó ninguno de los recursos mencionados la última vez que tuvo dificultad para respirar o hinchazón en los tobillos. Entre los que probaron alguno de los recursos, el 27,7% dijo que estaba absolutamente seguro de que el recurso le ayudó, el 23,2% dijo que estaba seguro, el 8,9% dijo que estaba poco seguro y el 9,8% dijo que no estaba seguro.

Para la subescala Confianza, las preguntas con mayor confianza fueron: “seguir el tratamiento recomendado” (45,5%), “evaluar la importancia de sus síntomas” (42,0%) y “evaluar si un medicamento funciona” (37,5%). La pregunta con menor confianza fue “estar libre de síntomas de IC” (23,2%).

La aplicación del Pictograma de Fatiga mostró que la mayoría de los participantes se sentía muy cansado (39,4%) o sumamente cansado (29,5%). La sensación de cansancio interfería en la ejecución de sus actividades diarias y, en consecuencia, en su calidad de vida, ya que el 49,0% afirmaba que, debido a la sensación incapacitante, podían hacer muy poco o solo lo que necesitaban hacer (20,5%) (Tabla 3).

Se evaluó una posible asociación entre las medidas de autocuidado y fatiga, y no encontramos resultados estadísticamente significativos entre la intensidad de la fatiga y las subescalas Mantenimiento (p = 0,266), Manejo (p = 0,074) y Confianza (p = 0,059) para el autocuidado. Se observaron resultados similares entre el impacto de la fatiga y las subescalas Mantenimiento (p = 0,834), Manejo (p = 0,609) y Confianza (p = 0,139) para el autocuidado de los participantes.

La distribución de las respuestas obtenidas en las subescalas Mantenimiento, Manejo y Confianza del autocuidado de los participantes, según la intensidad (pregunta 1) y el impacto (pregunta 2) de la fatiga se presentan, respectivamente, en las Figuras 1 y 2.

**Tabla 3-:** Evaluación de la fatiga de los 132 participantes, según el Pictograma de Fatiga. Ribeirão Preto, SP, Brasil, 2021-2022

**Pictograma de fatiga**	n (%)*
**Pregunta 1 – ¿En qué medida te has sentido cansado en la última semana?**	
Nada cansado	9 (6,8)
Un poco cansado	12 (9,1)
Moderadamente cansado	20 (15,2)
Muy cansado	52 (39,4)
Sumamente cansado	39 (29,5)
**Pregunta 2 – ¿En qué medida la sensación de cansancio te impide hacer lo que quieres hacer?**	
Puedo hacer todo lo que hago habitualmente	7 (5,3)
Puedo hacer casi todo lo que hago habitualmente.	9 (6,8)
Puedo hacer algunas de las cosas que hago habitualmente.	24 (18,2)
Solo hago lo que necesito hacer.	27 (20,5)
Puedo hacer muy poco	65 (49,2)

*n (%) = Número (porcentaje)

**Figura 1-:** Diagramas de caja con los resultados de las medidas de Mantenimiento, Manejo y Confianza para el autocuidado de la insuficiencia cardíaca, según la intensidad de la fatiga

**Figura 2-:** Diagramas de caja con los resultados de las medidas de Mantenimiento, Manejo y Confianza para el autocuidado de la insuficiencia cardíaca, según el impacto de la fatiga

## Discusión

El estudio tuvo como objetivo evaluar la asociación entre el autocuidado y la fatiga en pacientes hospitalizados por descompensación de IC durante la pandemia de COVID-19. Constatamos mantenimiento y manejo inadecuados del autocuidado en el mes previo a la hospitalización debido a la descompensación clínica de la IC. En la semana previa a la hospitalización, la mayoría de los participantes reportó fatiga intensa y compromiso de las actividades diarias. Sin embargo, no constatamos asociaciones estadísticamente significativas entre las subescalas de mantenimiento, manejo y confianza para el autocuidado de la IC y la intensidad de la fatiga y su impacto en las actividades diarias realizadas.

Dado que se trató de un estudio observacional, transversal que investigó una muestra no probabilística de 132 pacientes, consideramos que los resultados fueron de naturaleza exploratoria. Los resultados obtenidos no pueden generalizarse a todos los pacientes con las mismas características clínicas que fueron hospitalizados en Brasil durante la pandemia de COVID-19. Se trata de una muestra representativa y, aunque los participantes no fueron elegidos de forma aleatoria, representan la población que se estudiará. Los resultados obtenidos, así como los de otros estudios que utilizaron muestras de poblaciones clínicas^(12,32-33)^ aportan importantes contribuciones a la planificación de los estudios de intervención^(34-35)^, una vez que permitirán determinar el cálculo muestral y el tamaño muestral de estos estudios.

La interpretación de los resultados obtenidos debe considerarse según el contexto epidemiológico en que se realizó la investigación, en este caso los participantes fueron reclutados en un único servicio de salud (hospital público y docente del interior del estado de São Paulo), según las características demográficas que caracterizan a los pacientes atendidos por el Sistema Único de Salud y, principalmente, según el uso de instrumentos de medición elegidos entre otras opciones. El diseño transversal del estudio no permite inferir la asociación causa-efecto entre la fatiga y el autocuidado en la muestra estudiada. La declaración de la Organización Mundial de la Salud sobre el fin del estado de emergencia global de la pandemia de COVID-19 imposibilitará la realización de estudios de seguimiento de la población de interés, cuyo diseño prospectivo podría investigar la relación de las dos variables con la descompensación clínica de la IC.

Durante la pandemia de COVID-19, las acciones de autocuidado para las personas con IC disminuyeron^(27-30)^, con dificultades para acceder a los servicios de salud, interfiriendo en el seguimiento presencial de los pacientes. En Brasil, la baja adhesión al tratamiento farmacológico y dietético fueron los principales factores desencadenantes de la IC descompensada durante la pandemia de COVID-19, entre marzo y octubre del 2020^(40)^. Con la limitación de las consultas y el miedo al contagio^(20,25)^, los pacientes con IC disminuyeron la asistencia a los servicios de salud en Brasil^(41)^ y en otros países^(20,23,25,42-44)^, lo que contribuyó al aumento de la mortalidad^(44)^.

El perfil sociodemográfico de los participantes, con predominio de hombres, adultos mayores, casados o en unión estable, que no realizan trabajo remunerado, tienen bajos ingresos y poca escolaridad, también se observó en otros estudios nacionales e internacionales^(30,32,41,45)^.

Los resultados obtenidos también deben interpretarse teniendo en cuenta los instrumentos de medida elegidos para evaluar el autocuidado con IC^(5,37)^ y la fatiga en personas con este diagnóstico^(38-39)^. Las dos escalas permitieron comparar los resultados obtenidos durante el período de pandemia con otras investigaciones realizadas antes y durante la pandemia de COVID-19.

Al comparar los resultados obtenidos en las subescalas del SCHFI con los encontrados por investigadores brasileños, en estudios realizados antes de la pandemia, encontramos valores similares entre los pacientes atendidos en el consultorio ambulatorio del hospital donde realizamos nuestro estudio, que fueron seguidos en los primeros tres meses después del alta hospitalaria por descompensación de IC^(12)^. En otro estudio, también realizado en un hospital docente de la ciudad de São Paulo, con pacientes ambulatorios, los investigadores reportaron valores similares^(10)^. Nuestros resultados fueron similares a los observados por investigadores que usaron la misma herramienta para evaluar el autocuidado de pacientes en Estados Unidos de América^(27,29)^, Suiza^(18)^ y China^(45)^.

Respecto al síntoma de fatiga, en el grupo estudiado, los participantes reportaron la intensidad de la fatiga (muy o sumamente cansado) y su interferencia en la realización de las actividades diarias. La presencia del síntoma de fatiga en personas con IC está bien documentada^(32,46)^, afecta el desempeño de actividades físicas^(6)^, el autocuidado^(6-8,47)^ y la calidad de vida de los pacientes^(5,8)^. Durante la pandemia, se observó una reducción de la actividad física y un aumento de las manifestaciones de síntomas de descompensación de la IC como la fatiga^(26)^. Las personas que presentaban un mejor control de los síntomas antes de la pandemia fueron aquellas que mantuvieron un mejor autocuidado durante la pandemia en comparación con aquellas con un autocuidado deficiente^(28)^. Aunque hayamos encontrado pocos estudios que evaluaron la fatiga con el mismo instrumento^(6,32)^, nuestros resultados corroboran aquellos constatados en pacientes con IC, antes de la pandemia.

Los resultados obtenidos corroboran lo reportado por otros investigadores y refuerzan la necesidad de intervenciones de enfermería dirigidas a la alfabetización en salud para el autocuidado de estos individuos. Los estudios indican que la telemedicina desempeñó un papel importante durante el período pandémico al permitir el seguimiento, la identificación de pacientes prioritarios y la prestación de orientación en salud^(41,48-51)^. Esta modalidad de atención ya se empleaba antes de la pandemia, sin embargo no existía consenso entre los estudios respecto a su efectividad en pacientes con IC^(52)^. La pandemia fue un factor que impulsó la implementación a gran escala, mostrando ser una alternativa eficiente^(26,28,52)^.

La enfermería debe considerar el autocuidado en la IC como un proceso activo, que debe planificarse junto con el individuo y sus familiares, y que incluye desde el reconocimiento de los síntomas de descompensación clínica hasta la toma de decisión de buscar atención médica. Así, la educación para la salud pretende obtener resultados positivos en términos de adhesión a los tratamientos farmacológico y no farmacológico, favoreciendo el manejo, el mantenimiento y la confianza en el autocuidado de las personas con IC.

En cuanto a la contribución del estudio al avance del conocimiento científico sobre este tema, reforzamos la existencia de un autocuidado insatisfactorio de la IC en las cuatro semanas previas a la necesidad de hospitalizaciones no planificadas resultantes de la descompensación clínica de la enfermedad. Al comparar los resultados del presente estudio con los de estudios anteriores, observamos que, independientemente de la existencia de la pandemia de COVID-19, las personas con IC aún necesitan educación centrada en el autocuidado de su condición de salud, y el enfermero es uno de los profesionales más calificados para planificar esta compleja intervención, no solo para los pacientes sino también para sus familiares/cuidadores.

Las principales limitaciones del estudio fueron la imposibilidad de calcular el tamaño de la muestra determinando el número necesario de participantes para asegurar el carácter confirmatorio del resultado obtenido; el diseño transversal que no permite al investigador inferir sobre la causalidad de las variables, en este caso, autocuidado y fatiga; y el hecho de que el reclutamiento de los participantes se haya realizado en una sola institución hospitalaria.

## Conclusión

Durante la pandemia de COVID-19, las personas hospitalizadas debido a una descompensación clínica de la IC evaluaron su autocuidado en las cuatro semanas previas a la hospitalización como inadecuado para el Manejo, el Mantenimiento y el Conocimiento de la IC. La fatiga fue considerada intensa por la mayoría en la semana anterior a la hospitalización y afectó el desarrollo de las actividades diarias. Al verificar la presencia de una posible asociación entre autocuidado y fatiga, no constatamos resultados estadísticamente significativos en el grupo investigado.

## Data Availability

Los conjuntos de datos relacionados con este artículo estarán disponibles previa solicitud al autor correspondiente.
